# Impact of stress hyperglycemia ratio on acute kidney injury and mortality in patients with cardiogenic shock: a retrospective analysis

**DOI:** 10.3389/fendo.2025.1606819

**Published:** 2025-08-27

**Authors:** An Cai, Liqin Zhuang, Min He, Song Huang, Jinying Tong

**Affiliations:** ^1^ Department of Cardiovascular Medicine, People’s Hospital of Yingtan City, Yingtan, China; ^2^ Department of Cardiology, The First Affiliated Hospital of Guangxi Medical University, Nanning, China

**Keywords:** stress hyperglycemia ratio, cardiogenic shock, intensive care unit, acute kidney injury, mortality

## Abstract

**Aims:**

This study investigated the predictive value of the stress hyperglycemia ratio (SHR) for acute kidney injury (AKI) and mortality in cardiogenic shock (CS).

**Methods:**

A retrospective analysis was conducted on patients with CS from the Medical Information Mart for Intensive Care IV database based on SHR values. The primary outcome was AKI incidence, with in-hospital and 90-day mortality as secondary outcomes in the subgroup with AKI. Logistic regression assessed the relationship between SHR and AKI as well as in-hospital mortality, while Cox regression was employed to evaluate 90-day mortality. Restricted cubic spline curves were utilized to explore nonlinear associations.

**Results:**

Among 378 patients with CS, 56.9% developed AKI. Elevated SHR was associated with a higher risk of AKI (OR 2.58, 95% CI 1.44–4.81). In the AKI subgroup, SHR exhibited a U-shaped relationship with mortality (*P* for non-linearity < 0.05). An SHR above 1.26 was linked to increased in-hospital (OR 2.74, 95% CI 1.35–5.80) and 90-day mortality (HR 2.84, 95% CI 1.95–4.13).

**Conclusions:**

SHR is independently associated with both AKI and mortality in CS. A U-shaped curve suggests that optimal glycemic control may improve patient outcomes. Prospective studies are needed to validate these findings and further investigate SHR as a prognostic marker.

## 1 Introduction

Cardiogenic shock (CS) is a life-threatening condition characterized by significantly reduced cardiac output, severe hypotension, and inadequate end-organ perfusion, leading to persistently high morbidity and mortality despite advancements in diagnostics, pharmacotherapy, and early reperfusion strategies (1–3). CS most commonly complicates acute myocardial infarction (AMI), affecting approximately 5–10% of AMI cases, but can also arise from heart failure (HF), severe valvular diseases, or myocarditis (4). Among its many complications, acute kidney injury (AKI) poses a particularly formidable challenge. AKI exacerbates the risk of adverse clinical events and serves as a strong prognostic indicator, signaling a considerably worse outcome (5–7). In CS, the presence of AKI is associated with approximately a six-fold increase in in-hospital mortality risk, underscoring the critical importance of early identification of high-risk individuals (8). Early detection allows for timely intervention, potentially preventing irreversible organ damage and improving both immediate and long-term outcomes.

In critically ill patients, stress-induced hyperglycemia (SIH)—an acute spike in blood glucose triggered by physiological or psychological stress—has garnered significant attention as a key prognostic factor (9). However, simple admission blood glucose (ABG) values can be influenced by chronic dysglycemia, thus obscuring the true extent of acute metabolic disruptions. Beyond transient hyperglycemia, emerging evidence links SIH to both AKI and adverse cardiovascular outcomes, suggesting its potential role in shaping the clinical course of critically ill patients (10). Despite these insights, current CS management guidelines remain primarily focused on hemodynamic support and addressing underlying etiologies, offering limited guidance on glycemic control (11).

To address this gap, the stress hyperglycemia ratio (SHR) was introduced as a more nuanced measure of acute glycemic stress (12). By combining ABG with long-term glycemic indicators such as Hemoglobin A1c (HbA1c), SHR provides a relative assessment of acute hyperglycemia adjusted for baseline glycemic status. Elevated SHR has been consistently linked to poor outcomes in coronary artery disease, atrial fibrillation (AF), and HF (13–17). Furthermore, SHR has demonstrated superior predictive value compared to ABG alone for in-hospital and intensive care unit (ICU) mortality in CS (18), and recent studies have associated SHR with the incidence of AKI in critically ill patients (19). Whether this association specifically holds for patients with CS, who face a uniquely high risk for both cardiac and renal complications, remains uncertain. This study aims to clarify the relationship between SHR and AKI in patients with CS admitted to the ICU, potentially advancing early risk stratification, guiding targeted interventions, and improving patient outcomes.

## 2 Methods

### 2.1 Data source

This retrospective observational study utilized data from the Medical Information Mart for Intensive Care IV (MIMIC-IV; version 3.0, 2008–2022), a publicly available critical care database collected at Beth Israel Deaconess Medical Center, Boston, Massachusetts, USA (20). MIMIC-IV contains time-stamped clinical data for patients treated in the ICU. After completing the required Collaborative Institutional Training Initiative program, one author (Liqin Zhuang; certification no. 67404818) obtained authorized access to the database and extracted all relevant variables. All records in MIMIC-IV are fully anonymized. The study adheres to the principles of the Declaration of Helsinki and was approved by the relevant review board, which waived the requirement for individual informed consent due to the use of de-identified data.

### 2.2 Study population

**Figure 1:**
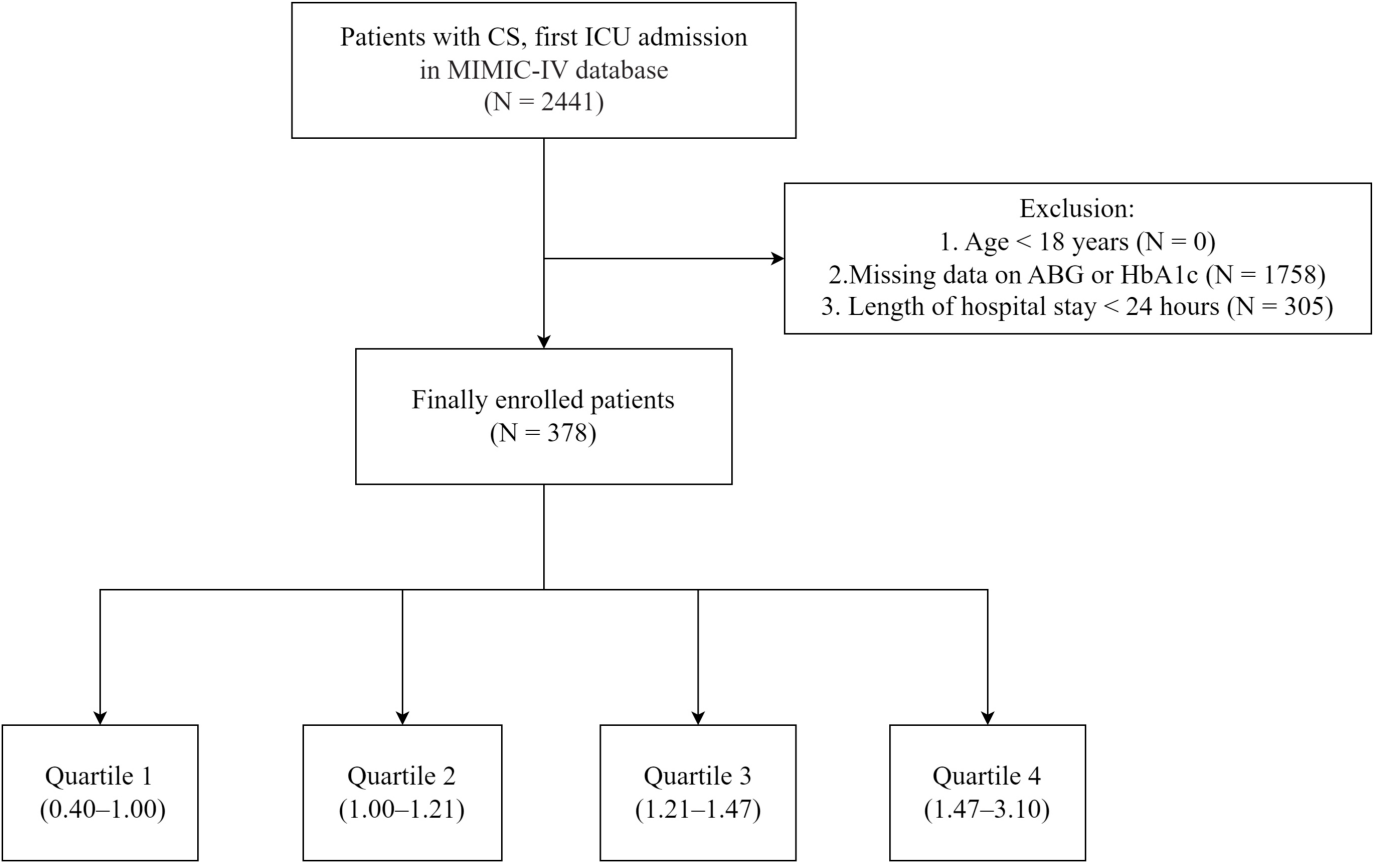
Flowchart of the study. MIMIC-IV, Medical Information Mart for Intensive Care IV; ICU, Intensive Care Unit; ABG, Admission Blood Glucose; HbA1c, Hemoglobin A1c; CS, Cardiogenic Shock; SHR, Stress Hyperglycemia Ratio.

Patients aged 18 years or older with a diagnosis of CS, admitted to the ICU, were included in the analysis. The diagnosis of CS was confirmed using relevant ICD-9/10 codes. AKI was defined according to widely accepted clinical practice guidelines, which include any of the following: an increase in serum creatinine by ≥ 0.3 mg/dL (or ≥ 26.5 μmol/L) within 48 hours; an increase in serum creatinine to ≥ 1.5 times the baseline value within the prior 7 days; or urine output ≤ 0.5 mL/kg/h for 6 hours (21). Only the first ICU admission was considered for patients with multiple admissions. Patients with hospitalizations of less than 24 hours (n = 305) or missing ABG or HbA1c measurements (n = 1,758) were excluded. After applying these criteria, the final cohort comprised 378 patients (**Figure 1**).

### 2.3 Data extraction

Data were extracted using structured query language *via* PostgreSQL (version 16.2) and Navicat Premium (version 16), retrieving information from the first 24 hours of ICU admission. Within this timeframe, demographic data and vital signs (age, gender, body mass index [BMI], blood pressure, heart rate, respiratory rate, oxygen saturation) were collected, along with severity of illness indices (Sequential Organ Failure Assessment [SOFA], Acute Physiology and Chronic Health Evaluation III [APACHE III], Systemic Inflammatory Response Syndrome, Oxford Acute Severity of Illness Score, Glasgow Coma Scale), and laboratory parameters (white blood cell count, platelets, red blood cell distribution width [RDW], electrolytes, ABG, HbA1c, coagulation profiles, bilirubin, blood urea nitrogen [BUN], and creatinine). Comorbidity information—atrial fibrillation (AF), cardiac arrest, hypertension, HF, AMI, chronic kidney disease (CKD), diabetes, old myocardial infarction (OMI), and dyslipidemia—was also recorded, along with intervention data (e.g., mechanical ventilation, percutaneous coronary intervention [PCI], coronary artery bypass grafting) and prescribed therapies (e.g., insulin, dobutamine, dopamine, epinephrine, norepinephrine) administered during hospitalization. Variables with more than 25% missing data were excluded, and missing values for other variables were imputed using a random forest approach to maintain data integrity. The SHR was calculated as [ABG (mg/dL)]/[28.7 × HbA1c (%) – 46.7] (12).

### 2.4 Outcome events

The primary outcome was the incidence of AKI in patients with CS during hospitalization. The secondary outcomes were in-hospital and 90-day mortality rates among CS patients who developed AKI.

### 2.5 Statistical analysis

Normality of continuous variables was assessed using the Shapiro–Wilk test. Continuous variables were summarized as mean ± standard deviation or median (interquartile range) based on distribution; categorical data were presented as frequencies (percentages). For group comparisons, chi-square or Fisher’s exact test was used for categorical variables, and Student’s t-test or Wilcoxon rank-sum test for continuous variables, as appropriate. Multicollinearity was assessed using the Variance Inflation Factor, with a threshold of 5 for excluding collinear predictors. Patients were stratified into four groups based on SHR values. Kaplan-Meier curves and log-rank tests were used to compare 90-day survival across SHR-defined groups. Multivariate logistic and Cox regression analyses were conducted to evaluate the relationships between SHR and AKI incidence, in-hospital mortality, and 90-day mortality, reporting odds ratios (ORs) or hazard ratios (HRs) with 95% confidence intervals (CIs). The unadjusted model included SHR only, while the fully adjusted model accounted for age, gender, BMI, platelets, potassium, total bilirubin, BUN, creatinine, AF, hypertension, CKD, and OMI. Restricted cubic spline (RCS) models were employed to assess nonlinear associations between SHR and the risk of AKI, in-hospital mortality, and 90-day mortality. Subgroup analyses examined variations in the SHR-AKI association by age (< 65 *vs*. ≥ 65 years), gender, and key comorbidities (AF, AMI, CKD, cardiac arrest, HF, hypertension, diabetes). All statistical analyses were conducted using R (version 4.3.3), with statistical significance set at a two-sided *P*-value < 0.05.

## 3 Results

### 3.1 Clinical characteristics at ICU admission

A total of 378 patients with CS were included, with a median age of 69.0 years (IQR, 60.0–78.0), and 63.0% (238/378) were male. The median SHR was 1.21 (1.00–1.47), and 56.9% (215/378) of patients developed AKI during hospitalization. Patients were stratified into quartiles based on SHR values: Q1 (0.40–1.00), Q2 (1.00–1.21), Q3 (1.21–1.47), and Q4 (1.47–3.10), with median SHR values of 0.87 (0.78–0.93), 1.12 (1.07–1.16), 1.30 (1.25–1.38), and 1.70 (1.57–1.93), respectively. Clinical characteristics at ICU admission across these quartiles were summarized in **Table 1**. The majority of patients with CS had a history of AMI or HF. Compared to lower SHR quartiles, patients in the Q4 group were younger, exhibited more severe illness at admission, and had higher rates of cardiac arrest and AMI. They also had elevated white blood cell counts, ABG, anion gap, and creatinine levels, along with increased use of insulin and dopamine (all *P* < 0.05).

**Table 1:** Clinical characteristics at ICU admission across SHR quartiles.

Variables	Quartile 1 (*N* = 95)	Quartile 2 (*N* = 95)	Quartile 3 (*N* = 94)	Quartile 4 (*N* = 94)	*P*
Demographics					
Age, years	69.0 [56.5;79.5]	71.0 [63.0;76.5]	69.5 [60.0;78.0]	68.0 [61.0;77.0]	0.748
Gender, male, n(%)	60 (63.2%)	52 (54.7%)	64 (68.1%)	62 (66.0%)	0.243
Body mass index, kg/m2	28.0 [24.0;30.8]	26.9 [24.7;31.1]	27.4 [24.8;31.7]	27.9 [24.1;33.3]	0.816
Vital signs					
Heart rate, bpm	86.0 [75.7;102]	85.0 [74.8;95.0]	83.9 [76.2;91.3]	89.9 [79.0;104]	0.033
Systolic blood pressure, mmHg	109 [102;116]	108 [102;115]	107 [101;114]	106 [101;112]	0.301
Diastolic blood pressure, mmHg	66.7 [61.4;73.1]	65.4 [60.3;71.0]	65.1 [60.1;71.6]	66.5 [59.8;73.4]	0.878
Scoring systems					
SOFA score	5.00 [3.00;8.00]	6.00 [2.50;9.00]	6.50 [4.00;9.00]	7.00 [5.00;10.0]	0.001
APACHE III score	42.0 [34.0;58.0]	46.0 [36.0;54.0]	44.0 [34.2;54.8]	56.0 [43.5;74.5]	<0.001
SIRS score	3.00 [2.00;3.00]	3.00 [2.00;3.00]	3.00 [3.00;3.00]	3.00 [3.00;4.00]	<0.001
OASIS score	31.0 [26.0;37.0]	34.0 [29.0;40.0]	33.5 [28.0;38.0]	37.0 [31.0;43.0]	<0.001
GCS score	15.0 [14.0;15.0]	15.0 [15.0;15.0]	15.0 [15.0;15.0]	15.0 [15.0;15.0]	0.564
Laboratory results					
White blood cell count, K/uL	10.6 [7.95;13.4]	13.6 [10.9;16.1]	14.2 [11.5;17.7]	15.8 [12.9;19.3]	<0.001
Platelets count, K/uL	188 [141;242]	199 [158;267]	196 [156;244]	210 [170;273]	0.090
Red blood cell distribution width, %	14.6 [13.6;16.1]	14.1 [13.4;15.2]	14.3 [13.4;15.5]	14.0 [13.3;15.1]	0.201
Serum sodium, mEq/L	139 [136;140]	138 [136;140]	138 [136;140]	138 [135;140]	0.439
Serum potassium, mEq/L	4.10 [3.86;4.38]	4.18 [3.91;4.56]	4.28 [4.02;4.55]	4.25 [3.93;4.70]	0.080
Admission blood glucose, mg/dL	116 [102;158]	134 [122;150]	156 [144;173]	214 [174;259]	<0.001
HbA1c, %	6.30 [5.70;9.35]	5.80 [5.50;6.25]	5.70 [5.50;6.00]	5.70 [5.20;6.20]	<0.001
Anion gap, mmol/L	14.5 [12.0;16.1]	14.5 [12.6;16.0]	15.0 [13.1;17.0]	16.6 [14.4;19.5]	<0.001
Partial thromboplastin time, s	51.3 [34.5;73.3]	49.0 [35.9;67.6]	52.5 [39.5;72.8]	56.7 [39.0;84.4]	0.193
Total Bilirubin, μmol/L	0.71 [0.50;1.22]	0.70 [0.50;1.17]	0.67 [0.45;1.04]	0.65 [0.40;0.99]	0.786
Blood urea nitrogen, mg/dL	25.0 [17.2;43.3]	24.5 [17.5;40.8]	25.4 [18.0;38.1]	26.7 [19.0;40.5]	0.675
Creatinine, μmol/L	1.19 [0.92;1.77]	1.20 [0.94;1.90]	1.38 [0.96;2.00]	1.52 [1.05;2.22]	0.032
SHR	0.87 [0.78;0.93]	1.12 [1.07;1.16]	1.30 [1.25;1.38]	1.70 [1.57;1.93]	<0.001
Comorbidities, n (%)					
Atrial fibrillation	40 (42.1%)	41 (43.2%)	47 (50.0%)	48 (51.1%)	0.491
Acute kidney injury:	45 (47.4%)	47 (49.5%)	58 (61.7%)	65 (69.1%)	0.006
Cardiac arrest:	8 (8.42%)	13 (13.7%)	16 (17.0%)	27 (28.7%)	0.002
Hypertension:	65 (68.4%)	78 (82.1%)	66 (70.2%)	69 (73.4%)	0.145
Heart failure	76 (80.0%)	70 (73.7%)	76 (80.9%)	69 (73.4%)	0.467
Chronic kidney disease	28 (29.5%)	23 (24.2%)	26 (27.7%)	22 (23.4%)	0.752
Acute myocardial infarction:	42 (44.2%)	53 (55.8%)	51 (54.3%)	66 (70.2%)	0.004
Old myocardial infarction:	18 (18.9%)	11 (11.6%)	10 (10.6%)	8 (8.51%)	0.146
Diabetes:	45 (47.4%)	36 (37.9%)	22 (23.4%)	40 (42.6%)	0.005
Ventilation	80 (84.2%)	84 (88.4%)	89 (94.7%)	82 (87.2%)	0.142
Medication					
Insulin	69 (72.6%)	63 (66.3%)	68 (72.3%)	79 (84.0%)	0.046
Dobutamine	17 (17.9%)	12 (12.6%)	21 (22.3%)	24 (25.5%)	0.130
Dopamine	14 (14.7%)	12 (12.6%)	10 (10.6%)	24 (25.5%)	0.025
Epinephrine	16 (16.8%)	18 (18.9%)	20 (21.3%)	27 (28.7%)	0.212
Norepinephrine	38 (40.0%)	45 (47.4%)	45 (47.9%)	54 (57.4%)	0.121
**In-hospital mortality**	17 (17.9%)	15 (15.8%)	24 (25.5%)	43 (45.7%)	<0.001
**90-day mortality**	29 (30.5%)	27 (28.4%)	27 (28.7%)	51 (54.3%)	<0.001

SHR, the stress hyperglycemia ratio; SOFA, the Sequential Organ Failure Assessment; APACHE III, the Acute Physiology and Chronic Health Evaluation III; SIRS, the systemic inflammatory response syndrome; OASIS, the Oxford Acute Severity of Illness Score; GCS, the Glasgow Coma Scale; HbA1c, glycated hemoglobin.

Bold values indicate section headers grouping related variables, not individual variables.

Additionally, **Supplementary Table S1** compared clinical characteristics at ICU admission between patients with and without AKI during hospitalization. Patients with AKI had a higher SHR (1.26 [1.03–1.54] *vs*. 1.15 [0.96–1.35], *P* = 0.002), were more frequently male, had higher BMI, and more commonly presented with AF, hypertension, CKD, OMI, diabetes, and required dobutamine and norepinephrine. They also had elevated RDW, potassium, glucose, anion gap, partial thromboplastin time, total bilirubin, BUN, and creatinine levels, along with reduced platelet counts (all *P* < 0.05). Furthermore, patients with AKI had higher SOFA and APACHE III scores (all *P* < 0.05).

### 3.2 Primary endpoint

**Figure 2:**
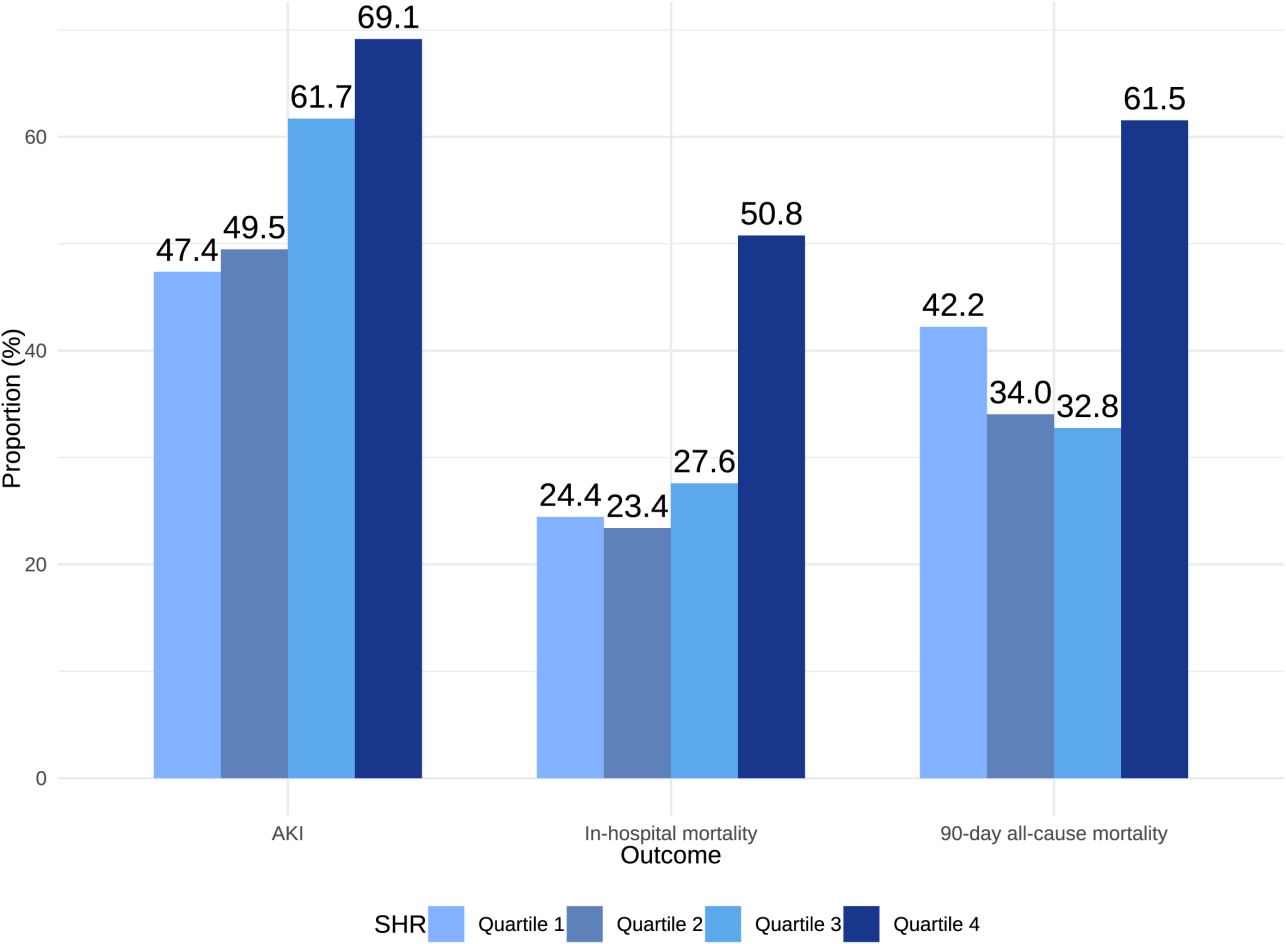
AKI incidence, in-hospital mortality, and 90-day mortality based on SHR quartiles (Q4). AKI, Acute Kidney Injury; SHR, Stress Hyperglycemia Ratio.

**Figure 3:**
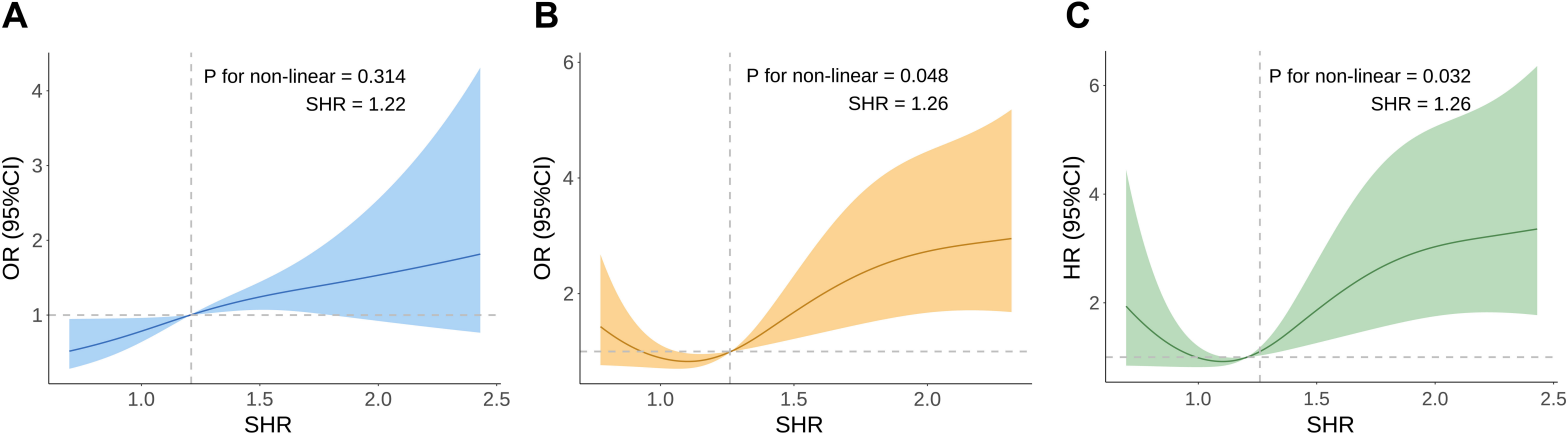
Restricted cubic spline curve for SHR with AKI incidence **(A)**, in-hospital mortality **(B)**, and 90-day mortality **(C)**. AKI, Acute Kidney Injury; SHR, Stress Hyperglycemia Ratio; OR, Odds Ratios; HR, Hazard Ratios; CI, Confidence Intervals.

A stepwise increase in AKI incidence was observed across SHR quartiles (Q1 *vs*. Q2 *vs*. Q3 *vs*. Q4: 47.4%, 49.5%, 61.7%, 69.1%; *P* = 0.006) (**Table 1**; **Figure 2**). As a continuous variable, SHR was significantly associated with AKI risk in both unadjusted (OR, 2.11; 95% CI, 1.27–3.63; *P* = 0.005) and fully adjusted models (OR, 2.58; 95% CI, 1.44–4.81; *P* = 0.002). When analyzed as a categorical variable, SHR quartiles Q3 and Q4 were significantly associated with AKI risk in both unadjusted (Q1 *vs*. Q2: OR, 1.09 [95% CI 0.62–1.93] *P* = 0.8; Q3: OR, 1.79 [95% CI 1.01–3.21] *P* = 0.049; Q4: OR, 2.49 [95% CI 1.38–4.55] *P* = 0.003) and fully adjusted models (Q1 *vs*. Q2: OR, 1.13 [95% CI 0.57–2.24] *P* = 0.73; Q3: OR, 2.25 [95% CI 1.13–4.55] *P* = 0.022; Q4: OR, 3.13 [95% CI 1.54–6.54] *P* = 0.002) (**Table 2**). The RCS curve demonstrated a dose-response relationship between SHR and AKI risk (*P* for non-linearity = 0.314) (**Figure 3A**). Moreover, a risk stratification analysis of SHR for the primary endpoint in subgroups by age, gender, AF, AMI, CKD, cardiac arrest, HF, hypertension, and diabetes showed consistent results, with no significant interactions (**Supplementary Figure S1**). Although the association between SHR and AKI appeared somewhat stronger in patients with diabetes, no statistically significant interaction was detected (*P* for interaction = 0.414), suggesting that the predictive value of SHR was broadly consistent irrespective of diabetes status.

**Table 2:** Associations of SHR with AKI incidence.

Categories	Unadjusted model		Adjusted model	
	OR (95% CI)	P-value	OR (95% CI)	*P*-value
Continuous variable per 1 unit	2.11 (1.27-3.63)	0.005	2.58 (1.44-4.81)	0.002
Quartile				
Q 1	Reference		Reference	
Q 2	1.09 (0.62-1.93)	0.8	1.13 (0.57-2.24)	0.73
Q 3	1.79 (1.01-3.21)	0.049	2.25 (1.13-4.55)	0.022
Q 4	2.49 (1.38-4.55)	0.003	3.13 (1.54-6.54)	0.002
*P* for trend	0.006		0.006	

Adjusted Model: adjusted for adjusted for age, gender, body mass index, platelets, potassium, blood urea nitrogen, total bilirubin, creatinine, atrial fibrillation, hypertension, chronic kidney disease, and old myocardial infarction.

SHR, the stress hyperglycemia ratio; OR, odds ratios; CI, confidence intervals.

### 3.3 Secondary endpoints

For in-hospital mortality, patients in Q4 exhibited the highest rate (50.8%) compared to Q1 (24.4%), Q2 (23.4%), and Q3 (27.6%) (*P* = 0.004) (**Figure 2**). SHR, when considered a continuous variable, was a significant predictor of in-hospital mortality in both unadjusted (OR, 2.87; 95% CI, 1.49–5.76; *P* = 0.002) and fully adjusted models (OR, 2.74; 95% CI, 1.35–5.80; *P* = 0.006). Categorical analyses similarly showed a significant association for Q4 relative to Q1 in both unadjusted (OR, 3.19; 95% CI, 1.41–7.59; *P* = 0.007) and fully adjusted models (OR, 3.09; 95% CI, 1.27–7.94; *P* = 0.015) (**Table 3**). The RCS curve further indicated a U-shaped relationship between SHR and in-hospital mortality (*P* for non-linearity = 0.048), with risk increasing when SHR surpassed 1.26 (**Figure 3B**).

**Table 3:** Associations of SHR with in-hospital and 90-day mortality and in patients with CS and AKI.

Categories	Unadjusted model		Adjusted model	
	OR (95% CI)	*P*-value	OR (95% CI)	*P*-value
In-hospital mortality				
Continuous variable per 1 unit	2.87 (1.49-5.76)	0.002	2.74 (1.35-5.80)	0.006
Quartile				
Q 1	Reference		Reference	
Q 2	0.94 (0.36-2.48)	>0.9	1.09 (0.39-3.08)	0.87
Q 3	1.18 (0.49-2.93)	0.7	1.34 (0.52-3.58)	0.55
Q 4	3.19 (1.41-7.59)	0.007	3.09 (1.27-7.94)	0.015
*P* for trend	0.004		0.004	

	HR (95% CI)	P-value	HR (95% CI)	P-value
90-day mortality				
Continuous variable per 1 unit	2.02 (1.34-3.05)	<0.001	2.84 (1.95-4.13)	<0.001
Quartile				
Q 1	Reference		Reference	
Q 2	0.79 (0.40-1.53)	0.5	0.83 (0.42-1.66)	0.61
Q 3	0.77 (0.41-1.46)	0.4	0.93 (0.48-1.80)	0.82
Q 4	1.92 (1.11-3.32)	0.019	2.14 (1.18-3.89)	0.012
*P* for trend	<0.001		<0.001	

Adjusted Model: adjusted for adjusted for age, gender, body mass index, platelets, potassium, blood urea nitrogen, total bilirubin, creatinine, atrial fibrillation, hypertension, chronic kidney disease, and old myocardial infarction.

SHR, the stress hyperglycemia ratio; OR, odds ratios; HR, hazard ratios; CI, confidence intervals.

**Figure 4:**
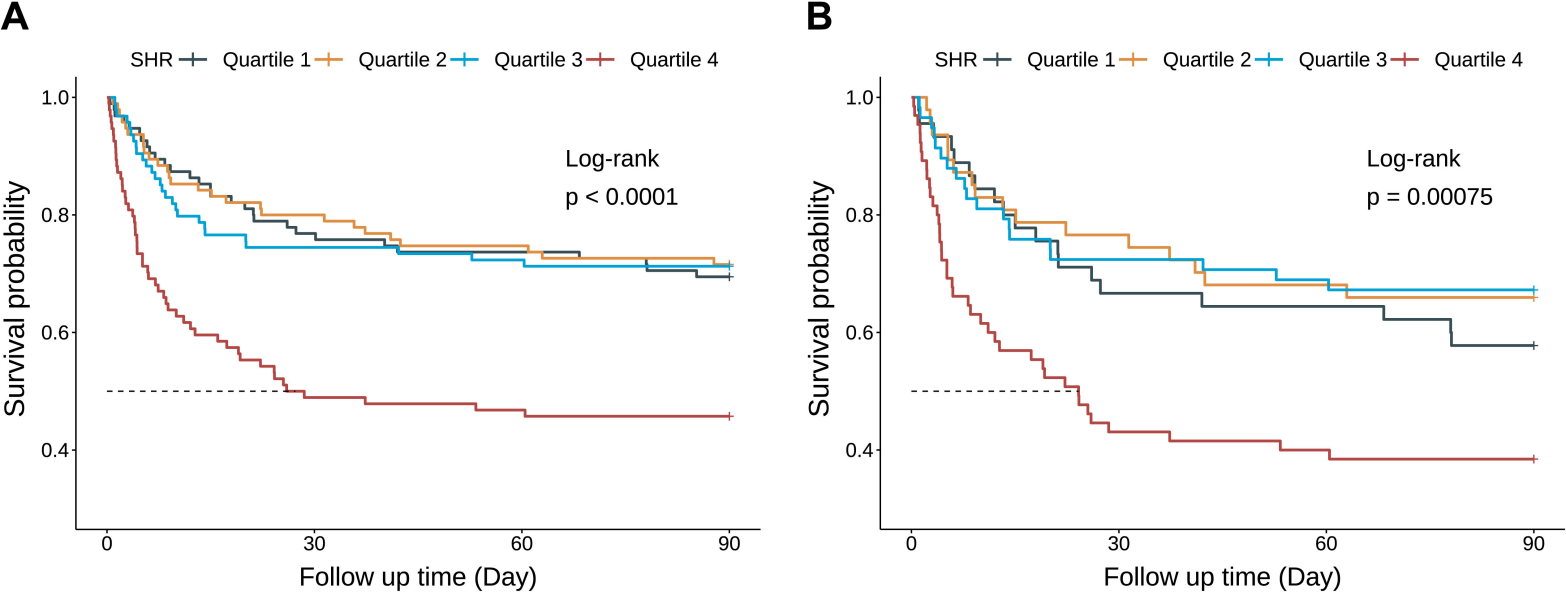
Kaplan-Meier survival analysis curves for 90-day mortality among the entire study CS cohort **(A)** and the AKI subgroup **(B)**. *P*-values reflect log-rank comparisons versus Quartile 1 (reference group). SHR, Stress Hyperglycemia Ratio.

Kaplan-Meier curves revealed that patients in Q4 had the highest 90-day mortality in the overall CS cohort (**Figure 4A**) and the AKI subgroup (both Log-rank *P* < 0.001) (**Figure 4B**), but no such difference was observed in the non-AKI subgroup (Log-rank *P* = 0.18) (**Supplementary Figure S2**). Among patients with CS and AKI, those in the highest SHR quartile (Q4) had the highest 90-day mortality (Q4: 61.5% *vs*. Q1: 42.2%, Q2: 34.0%, Q3: 32.8%; *P* = 0.005) (**Figure 2**). As a continuous variable, SHR was significantly associated with 90-day mortality in both the unadjusted (HR, 2.02; 95% CI, 1.34–3.05; *P* < 0.001) and fully adjusted models (HR, 2.84; 95% CI, 1.95–4.13; *P* < 0.001). When analyzed categorically, Q4 showed a significant association with higher 90-day mortality compared to Q1 in both unadjusted (HR, 1.92; 95% CI, 1.11–3.32; *P* = 0.019) and fully adjusted models (HR, 2.14; 95% CI, 1.18–3.89; *P* = 0.012) (**Table 3**). The RCS curve demonstrated a U-shaped relationship (*P* for non-linearity = 0.032), with mortality risk increasing once SHR exceeded 1.26 (**Figure 3C**), suggesting that maintaining SHR levels below this inflection point may be associated with lower mortality.

## 4 Discussion

This study represents the first comprehensive investigation of the association between SHR and AKI in patients with CS, demonstrating that elevated SHR values are significantly associated with increased AKI incidence and subsequent mortality in this population. A dose-response relationship was identified between SHR and AKI incidence, while a U-shaped association was observed between SHR and both in-hospital and 90-day mortality, with a critical inflection point at an SHR of 1.26. Patients with either markedly elevated or exceptionally low SHR values faced significantly higher mortality risks, and increased SHR levels were linked to a heightened susceptibility to AKI during hospitalization. These findings suggest that SHR may serve as a valuable prognostic marker in this patient population.

CS is characterized by severely diminished cardiac output, inadequate organ perfusion, and sustained tissue hypoxia (18). Despite advancements in prevention, diagnosis, and treatment, in-hospital mortality rates remain as high as 50% (5). While various prognostic factors have been identified, AKI has emerged as a key determinant of poor outcomes in CS. However, epidemiological data on AKI in CS remains limited, primarily due to the predominance of AMI cohorts, where AMI is a leading cause of CS (7). One prior study suggests that AKI incidence in CS could approach 60% (5), and MIMIC database analyses have indicated a rate of approximately 54% (22, 23). The 56.9% incidence in our study aligns closely with these estimates. AKI in these patients is associated with extended hospital stays, increased complication rates, and higher in-hospital mortality, particularly when severe renal impairment requires dialysis (6). Despite the exploration of risk assessment tools like the SOFA score (24), there remains a need for more accurate and practical biomarkers to identify high-risk patients early.

In critically ill settings, SIH has gained recognition as an indicator of metabolic stress. SIH refers to an acute rise in blood glucose in response to physiological or psychological challenges. However, ABG alone may not effectively differentiate acute hyperglycemia from chronic dysglycemia. By combining ABG with long-term glycemic control indicators such as HbA1c, SHR provides a relative measure of glycemic stress that accounts for baseline metabolic status (12). Several studies have demonstrated SHR’s predictive ability for AKI risk across diverse clinical settings. For instance, SHR outperformed ABG in predicting AKI in patients with diabetic AMI (10) and was identified as an independent AKI risk factor in critically ill diabetic patients (19). Additionally, Li et al. highlighted SHR as a significant risk factor for contrast-induced AKI in a large cohort of 19,965 patients undergoing coronary angiography (25), and Shan et al. observed a strong association between SHR and contrast-induced AKI in 3,137 patients undergoing coronary angiography or PCI (26). SHR has also been linked to AKI in patients with HF (27). Our findings that SHR closely correlates with AKI incidence in patients with CS extend the prognostic utility of SHR to this particularly vulnerable group.

SIH is prevalent in the ICU, affecting at least 50% of patients within the first 48 hours of admission (28). Unlike simple glucose measurements, SHR provides a more accurate reflection of SIH severity by capturing both acute and chronic glycemic components. Previous research has identified SHR as a potential prognostic marker for mortality in critically ill patients with AKI (29). In the present study, SHR was not only associated with AKI incidence but also exhibited a U-shaped relationship with both in-hospital and 90-day mortality in patients with CS. Similar U-shaped patterns have been observed in coronary artery disease, AF, HF, and sepsis (14, 30–32), suggesting that both severe hyperglycemia and pronounced hypoglycemia can be harmful. However, most prior studies focused on non-CS populations with different metabolic and hemodynamic profiles. In contrast, patients with CS face profound circulatory insufficiency and heightened susceptibility to renal hypoperfusion, potentially magnifying the adverse impact of dysregulated glycemia. This may indicate a unique interplay between acute metabolic stress and compromised organ perfusion in CS, underscoring the importance of maintaining balanced glycemic levels while accounting for nutritional and frailty status. Extremely low SHR values may result from insulin overuse, prolonged fasting, or inadequate nutrient intake (33, 34), and could also reflect underlying frailty or chronic malnutrition that compromises metabolic reserve and increases vulnerability to stressors (35). Therefore, careful glycemic management tailored to these specific risks may be crucial for improving renal outcomes and overall prognosis in CS.

Our findings suggest that elevated SHR indicates a state of increased metabolic and hemodynamic vulnerability in CS, thereby elevating the risk of AKI and all-cause mortality. Prolonged CS or AKI can result in irreversible organ damage, a hallmark of cardiorenal syndrome (36). Within this syndrome, SIH exacerbates hemodynamic and neurohormonal disturbances, along with inflammatory responses, further impairing renal perfusion and function (7). Acute hyperglycemia induces osmotic diuresis and intravascular volume depletion (37), amplifies inflammation and oxidative stress (38), and activates stress pathways mediated by the hypothalamic-pituitary-adrenal axis and sympathetic-adrenal system (39). These responses trigger cytokine surges, oxidative stress, diminished nitric oxide bioavailability, and endothelial damage (40). Concurrently, abnormal neurohormonal and inflammatory signals constrict the renal vasculature, reducing perfusion (41).

Under these adverse conditions, SIH has been shown in both experimental and clinical studies to drive mitochondrial dysfunction, oxidative stress, and endoplasmic reticulum (ER) stress—processes that include hyperglycemia-induced mitochondrial reactive oxygen species (ROS) production, impaired mitophagy, and activation of ER stress-mediated apoptotic pathways within renal tubular cells—ultimately leading to tubular epithelial injury (42–44). Disruptions in nitric oxide balance, excessive reactive oxygen species, and inhibited mitophagy exacerbate mitochondrial damage and endothelial dysfunction, increasing renal vulnerability (43, 45). Additionally, SIH fosters the formation of advanced glycation end-products, undermining microvascular integrity and promoting glomerulosclerosis (46). These interconnected mechanisms transform transient metabolic disturbances into persistent renal impairment, suggesting that elevated SHR, as a marker of disproportionate glycemic response, may reflect heightened susceptibility within the cardiorenal axis and highlight the potential role of SIH-driven processes, such as mitochondrial ROS production and ER stress, in AKI development among patients with CS.

From a clinical perspective, SHR integrates both chronic and acute glycemic measures, offering a practical and widely applicable tool to refine prognostic assessments. Given the routine availability of ABG and HbA1c tests, SHR can be easily incorporated into standard clinical workflows. In our study, an apparent inflection point was observed at approximately SHR = 1.26, beyond which the risk of mortality increased notably. This threshold may serve as a useful reference for identifying patients who could benefit from closer glycemic surveillance and more individualized metabolic management. However, whether glycemic interventions targeting specific SHR thresholds, such as adjusting insulin dosage or enhancing monitoring when SHR exceeds 1.26, can improve outcomes remains unclear. Although various studies suggest different cutoff values, evidence is lacking on whether SHR-guided glycemic interventions can improve clinical outcomes. Future prospective investigations are needed to determine whether SHR thresholds can serve as effective targets for optimizing glycemic control in patients with CS. The observed U-shaped association highlights the importance of maintaining balanced glycemic levels, as both excessive hyperglycemia and relative hypoglycemia appear to be linked with adverse outcomes. Additionally, SHR may complement established severity scores such as SOFA and APACHE III by incorporating metabolic stress dimensions not fully captured by traditional physiological parameters, potentially refining early risk stratification in patients with CS. Further prospective studies are needed to clarify the optimal glycemic targets in this population, to evaluate whether maintaining SHR below this inflection point can improve renal and overall outcomes, and to determine the incremental prognostic value of integrating SHR into existing models.

However, several limitations must be considered. First, as a single-center, retrospective, observational study with a limited sample size, causality cannot be established. Specifically, the retrospective design precludes clear determination of the temporal sequence between elevated SHR and worsening clinical outcomes, thereby preventing us from discerning whether higher SHR is a cause or merely a consequence of greater CS severity. Prospective validation in larger, multicenter cohorts is essential to confirm these findings and extend their applicability. Second, reliance on MIMIC database data may introduce selection bias. In particular, the exclusion of a large number of patients due to missing ABG or HbA1c measurements represents an important source of potential bias, as it may have resulted in a cohort more likely to undergo intensive glucose monitoring, thereby limiting the generalizability of our findings. Additionally, certain confounders—such as nephrotoxic drugs or detailed fluid management practices—were not fully captured. Third, the critical illness severity of these patients with CS may limit the generalizability of the results to less severe clinical contexts. Although most patients in our cohort had AMI or HF, detailed data on the exact primary etiology of CS were not available, which precluded more granular analyses by CS subtype. Moreover, the SHR cutoff value of 1.26 was identified from exploratory analysis within our cohort, and its applicability to broader patient populations requires external validation. Future large-scale, randomized trials are needed to clarify the mechanisms linking SHR and AKI in CS and to identify specific biomarkers or therapeutic targets. Ultimately, establishing SHR as a reliable prognostic marker could facilitate more personalized, data-driven clinical decision-making, improving outcomes for patients facing the challenges of CS-associated AKI.

## 5 Conclusion

In conclusion, this study demonstrates an association between SHR and AKI in patients with CS. Elevated SHR values are significantly associated with increased AKI incidence and subsequent mortality in patients with CS, highlighting the prognostic value of SHR for identifying high-risk individuals and underscoring the critical importance of maintaining optimal glycemic control in this clinical setting. Future research should focus on larger, multicenter, prospective studies to validate these findings.

## Data Availability

The raw data supporting the conclusions of this article will be made available by the authors, without undue reservation.
