# Supplementary material for: Impact of stress hyperglycemia ratio on acute kidney injury and mortality in patients with cardiogenic shock: a retrospective analysis
Source: Front Endocrinol (Lausanne). 2025 Aug 27;16:1606819. doi: 10.3389/fendo.2025.1606819 (PMC12420316; doi:10.3389/fendo.2025.1606819)
Supplement: Supplementary file 1 [file DataSheet1.docx]

Supplementary Material

# Supplementary Tables

Table S1. Baseline characteristics between patients with AKI and those without AKI.

| **Variables** | **Non-AKI** | **AKI** | ***P*** |
| --- | --- | --- | --- |
|  | ***N=163*** | ***N=215*** |  |
| **Demographics** |  |  |  |
| Age, years | 68.0 [59.5;75.0] | 71.0 [60.5;80.0] | 0.104 |
| Gender, male, n(%) | 90 (55.2%) | 148 (68.8%) | 0.009 |
| Body mass index, kg/m2 | 27.2 [24.0;30.1] | 27.7 [24.8;32.7] | 0.029 |
| **Vital signs** |  |  |  |
| Heart rate, bpm | 85.0 [75.6;96.5] | 86.0 [75.8;100] | 0.420 |
| Systolic blood pressure, mmHg | 107 [102;116] | 107 [101;114] | 0.670 |
| Diastolic blood pressure, mmHg | 66.5 [60.8;72.2] | 65.0 [59.8;72.0] | 0.373 |
| **Scoring systems** |  |  |  |
| SOFA score | 5.00 [2.50;8.00] | 7.00 [4.00;9.00] | <0.001 |
| APACHE.III score | 39.0 [30.5;51.0] | 52.0 [41.0;65.0] | <0.001 |
| SIRS score | 3.00 [2.00;3.00] | 3.00 [2.00;3.00] | 0.294 |
| OASIS score | 33.0 [28.0;38.0] | 34.0 [28.0;41.0] | 0.078 |
| GCS score | 15.0 [15.0;15.0] | 15.0 [15.0;15.0] | 0.605 |
| **Laboratory results** |  |  |  |
| White blood cell count, K/uL | 13.5 [10.1;16.4] | 13.6 [10.8;17.7] | 0.218 |
| Platelets count, K/uL | 212 [172;269] | 191 [146;247] | 0.002 |
| Red blood cell distribution width, % | 13.9 [13.2;15.2] | 14.3 [13.6;15.5] | 0.010 |
| Serum sodium, mEq/L | 139 [136;140] | 138 [135;140] | 0.052 |
| Serum potassium, mEq/L | 4.10 [3.86;4.38] | 4.27 [4.00;4.69] | 0.003 |
| Admission blood glucose, mg/dL | 143 [118;180] | 162 [132;217] | <0.001 |
| HbA1c, % | 5.80 [5.45;6.60] | 5.80 [5.50;6.50] | 0.722 |
| Anion gap, mmol/L | 14.0 [12.0;15.8] | 15.7 [13.7;18.0] | <0.001 |
| Partial thromboplastin time, s | 49.9 [37.7;75.5] | 51.5 [36.4;70.1] | 0.949 |
| Total Bilirubin, μmol/L | 0.60 [0.40;0.95] | 0.74 [0.50;1.35] | 0.008 |
| Blood urea nitrogen, mg/dL | 18.6 [15.0;26.6] | 32.7 [23.0;50.3] | <0.001 |
| Creatinine, μmol/L | 0.95 [0.77;1.20] | 1.64 [1.27;2.20] | <0.001 |
| SHR, % | 1.15 [0.96;1.35] | 1.26 [1.03;1.54] | 0.002 |
| **Comorbidities, n (%)** |  |  |  |
| Atrial fibrillation | 65 (39.9%) | 111 (51.6%) | 0.030 |
| Cardiac arrest: | 25 (15.3%) | 39 (18.1%) | 0.561 |
| Hypertension: | 107 (65.6%) | 171 (79.5%) | 0.004 |
| Heart failure | 119 (73.0%) | 172 (80.0%) | 0.140 |
| Chronic kidney disease | 27 (16.6%) | 72 (33.5%) | <0.001 |
| Acute myocardial infarction: | 93 (57.1%) | 119 (55.3%) | 0.821 |
| Old myocardial infarction: | 28 (17.2%) | 19 (8.84%) | 0.023 |
| Diabetes: | 53 (32.5%) | 90 (41.9%) | 0.080 |
| Ventilation: | 146 (89.6%) | 189 (87.9%) | 0.733 |
| **Medication** |  |  |  |
| Insulin: | 111 (68.1%) | 168 (78.1%) | 0.037 |
| Dobutamine: | 16 (9.82%) | 58 (27.0%) | <0.001 |
| Dopamine: | 25 (15.3%) | 35 (16.3%) | 0.916 |
| Epinephrine: | 33 (20.2%) | 48 (22.3%) | 0.718 |
| Norepinephrine: | 67 (41.1%) | 115 (53.5%) | 0.022 |
| **In‐hospital mortality** | 28 (17.2%) | 71 (33.0%) | 0.001 |
| **90-day mortality** | 40 (24.5%) | 94 (43.7%) | <0.001 |

Abbreviations: SHR: the stress hyperglycemia ratio; SOFA: the Sequential Organ Failure Assessment; APACHE III: the Acute Physiology and Chronic Health Evaluation III; SIRS: the systemic inflammatory response syndrome; OASIS: the Oxford Acute Severity of Illness Score; GCS: the Glasgow Coma Scale; HbA1c: glycated hemoglobin.

# Supplementary Figures

Figure S1. Forest plots of odds ratios for the primary endpoint in different subgroups.


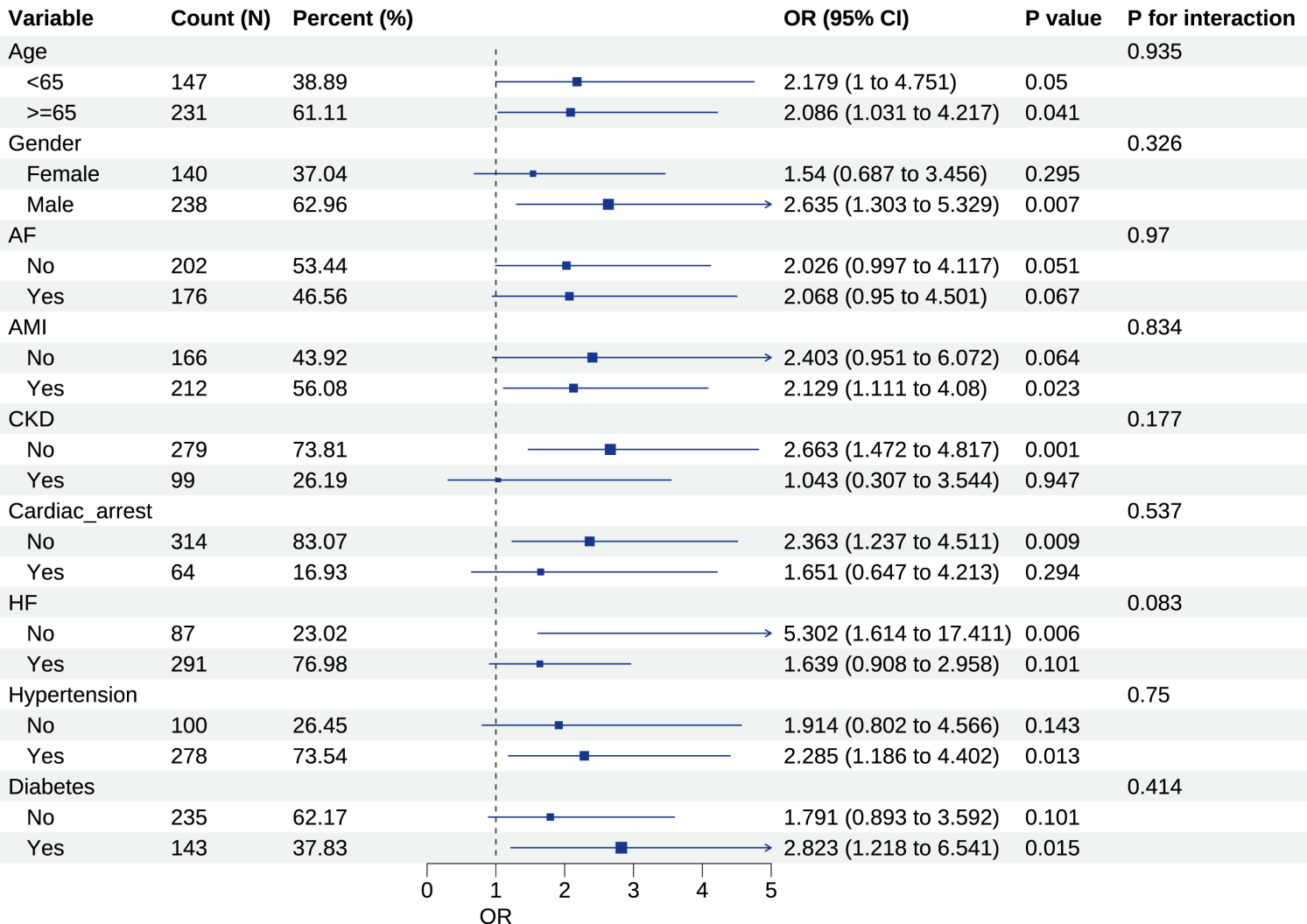


Abbreviations: AF: atrial fibrillation; AMI: acute myocardial infarction; CKD: chronic kidney disease; HF: heart failure; CI: confidence intervals

Figure S2. Kaplan–Meier survival analysis curves for 90-day mortality on the non-AKI subgroup.


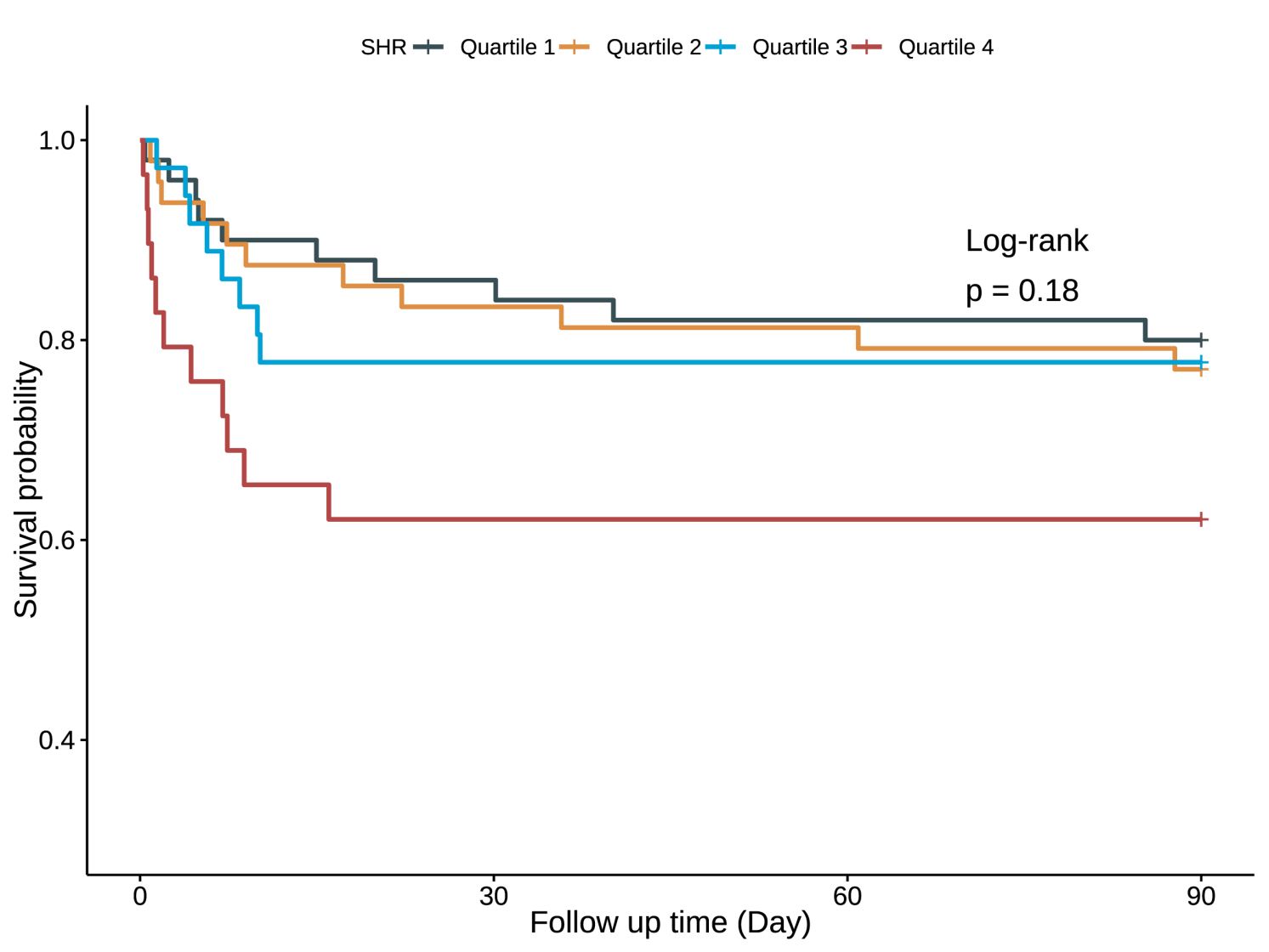


Abbreviations: SHR: Stress Hyperglycemia Ratio.
